# *Genista tridentata* L.: A Rich Source of Flavonoids with Anti-Inflammatory Activity

**DOI:** 10.3390/medicines7060031

**Published:** 2020-05-30

**Authors:** Diana C. G. A. Pinto, Mark A. M. Simões, Artur M. S. Silva

**Affiliations:** LAQV-REQUIMTE & Department of Chemistry, University of Aveiro, Campus de Santiago, 3810-193 Aveiro, Portugal; mark.simoes@outlook.com (M.A.M.S.); artur.silva@ua.pt (A.M.S.S.)

**Keywords:** *Genista tridentata*, *Pterospartum tridentatum*, isoflavones, flavonols, anti-inflammatory, genistein, biochanin A, rutin, daidzein

## Abstract

**Background:***Genista tridentata* L. is an endemic species from the Iberian Peninsula used in Portuguese traditional medicine to treat inflammation-related diseases; this and other health-promoting effects are usually associated with the flavonoids produced by this species. In fact, anti-inflammatory properties were established for several of these flavonoid derivatives. **Methods:** A careful survey of the reported data, using mainly the Scopus database and *Genista tridentata* and *Pterospartum tridentatum* as keywords, was done. We have examined the papers involving the plant and those about the most relevant flavonoids anti-inflammatory activity. **Results:** The literature survey demonstrates that species are used to treat several health problems such as antihyperglycemia, hypertension, and inflammatory episodes. It was also possible to establish its richness in flavonoid derivatives, from which several are potential anti-inflammatory agents. **Conclusions:** From our described and discussed analysis, it can be concluded that *Genista tridentata* is an excellent source of bioactive flavonoids. Moreover, its traditional use to treat inflammation episodes may be due to its flavonoid content, from which genistein, biochanin A, rutin, and daidzein can be emphasized.

## 1. Introduction

Inflammation is a natural defense mechanism involved in the body’s healing process, in which the body is protected from pathogens or abnormal cells [1]. However, if the inflammation is prolonged in time or serious, it can damage the healthy tissues and cause several diseases, such as cancer [2], Alzheimer’s and Parkinson’s diseases [3]. Therefore, the development of new anti-inflammatory drugs is still a demand, and plant secondary metabolites are considered a priority—in particular, those found in medicinal plants [4].

Among the plants used in Portuguese traditional medicine, *Genista tridentata* L. can be highlighted due to the important applications reported [5]; in fact, the plant, locally named carqueja, is in several regions called the “plant that heals everything” [5], and among its applications is the use to treat inflammatory diseases [6].

Flavonoids, a large family of natural compounds, are usually associated with anti-inflammatory activity [7], and most recently, we demonstrated that *G. tridentata* is rich in flavonoid derivatives [8], including some for which anti-inflammatory activities have been described. As examples, genistein, daidzein [9,10], and biochanin A [11,12] can be highlighted.

The most promising anti-inflammatory flavonoids that can be isolated from *G. tridentata* will be discussed in this review, emphasizing their mode of action and in vivo studies. Hopefully, this will help the scientific community to understand their involvement in inflammatory processes and consequently endorse the design for novel derivatives. Furthermore, the traditional medicine applications of *G. tridentata* will also be addressed and discussed. To accomplish this survey, we used mainly the Scopus database (69 articles), but also Web of Science (61 articles) and PubMed, mostly for the anti-inflammatory activity. The keywords used were the accepted name (*Genista tridentata*), the most common synonym (*Pterospartum tridentatum*), and also the less common one (*Chamaespartium tridentatum*). Naturally, in the survey there were also the flavonoid names combined with anti-inflammatory activity. Relevance was given to the most recent biological evaluations and the in vivo studies and the clinical trials. In all cases, the papers involving both the plant and the most relevant flavonoids anti-inflammatory activities.

## 2. *Genista tridentata*: Traditional Applications and Biological Activities

*Genista tridentata* L. is a bush endemic to the Iberian Peninsula where it grows wildly. Unfortunately, its taxonomy is a little controversial, and consequently, the literature survey is more complicated. The most found scientific name is *Pterospartum tridentatum* (L.) Willk., which is considered by some taxonomists [13] as the correct name, but other authors used *Chamaespartium tridentatum* (L.) P.E. Gibbs [14]. However, according to the Plant List database [15], these are synonyms of *Genista tridentata* L. and there are eleven other synonyms and three infraspecific *taxa* [15]. However, in our survey, only the abovementioned synonyms were found—it seems that the other synonyms and infraspecific *taxa* are not used in articles involving chemical profile and/or anti-inflammatory evaluations. Although we used all names in the literature survey, herein, we will refer the species by the accepted name reported in the Plant Lista database [15].

*Genista tridentata* is an Angiosperm belonging to the Leguminosae family [15], which grows spontaneously under Mediterranean thermal conditions, where it is known as carqueja [16]. *G. tridentata* is a perennial shrub that can reach up to one meter in height, with stems of woody and rigid consistency. The roots are well-liked and quite long and sometimes intertwine in the roots of other companion species. The stems are woody, erect or prostrate with laterally winged branches, forming false leaves of dark green color, cut out and of coriaceous consistency. Thus the branches have a flattened shape with two or three wing-shaped expansions, with an articulated appearance, ending with two or three teeth. The leaves, persistent, alternating, unifoliolate and triangular, appear to be tridentate, by the leaflets being united to the stipulations. The flowers are of an intense yellow and are arranged in corymbiform inflorescences, in groups of 3 to 10, gathered in small and tight bouquets. They have an induction in the sepals that line them. The fruit is an oblong-linear pod 10 to 12 mm long [17].

Despite the abovementioned disagreement in the *G. tridentata* taxonomy, the vernacular designation, carqueja, is referred to in the ethnopharmacological surveys. Consequently, it is possible to mention here that *G. tridentata* is used in the Iberian Peninsula, particularly in Portugal, in traditional medicine, mainly to treat influenza, cold, cough, stomach troubles, and nervousness, and is also used as a tonic, hepatic protector, sedative, cicatrizant, and diuretic [6,14,18,19]. In these applications, the population mainly uses extracts of the plant flowers, leaves, or the aerial parts. Consequently, it is suggested that the plant presents several therapeutic properties, from which antispasmodic, antihypertensive, and anti-inflammatory properties can be emphasized [6,14].

The flowers are used in folk medicine for the treatment of various disorders, including those relating to the respiratory system, digestive tract, nervous system, urinary system and dermatology; it has also been indicated for diabetes control [16,20] and is sometimes used in mixtures with other plants for this purpose [20]. Some authors referred to the use of *P. tridentatum* for the treatment of colds, stomach pains, intestinal problems, kidney disease, liver and gallbladder problems and also for rheumatism [21]. It was also indicated for pneumonia, bronchitis and tracheitis, headaches, cough, for low blood pressure levels and high levels of cholesterol, diabetes and even in weight loss programs. This species is known for its diuretic, purgative, laxative, hypotensive, hypoglycemic effects, and for its digestive properties [14,22]. The infusion of dried flowers is considered an excellent emollient [21].

One vital point that should be herein mentioned is the obligation to have scientific validations of the claimed properties, an aspect that it is not at all strange to the scientific community [23]. In this regard, several evaluation studies involving *G. tridentata* extracts were reported and will be herein presented and discussed. Most of the studies were performed using the flowers or the aerial parts extracted with polar solvents and in vitro antioxidant evaluations (Table 1).

The authors achieved the extract antioxidant activity index or antioxidant potential through several assays, from which DPPH^●^ (2,2-diphenyl-1-picrylhydrazyl radical) scavenging assay and β-carotene bleaching test are the most common. However, it is interesting to note that some authors used other less common tests, such as lipid peroxidation inhibition, through the decrease in TBARS (thiobarbituric acid reactive substances) [26,29,30,31], and, more recently, the oxidative hemolysis inhibition assay [31]. These diversifications in the assays are, in our opinion, very good because they can establish in more detail the *G. tridentata* health-promoting potential. Altogether, the reported results show that this species presents moderate to strong antioxidant activity, and apparently, the flower extracts and the water extracts are more active [25,27].

Another interesting feature in these reports is the fact that all authors obtained the total phenolic content and/or the total flavonoid content, and some established the polyphenolic profile or identified some of the phenolic compounds present [25,28,29,30,31]. In doing so, they associated the antioxidant activity to the polyphenolic content. On the other hand, some aspects of these reports are less enthusiastic, since the reported values are in different units; the positive controls used are different, making it impossible to perform comparisons.

Other evaluations, such as antifungal [32], antibacterial [31,33] agents, cytotoxicity activity in tumor and non-tumor cells [31], and even the immunostimulatory activity of the *G. tridentata* polysaccharides [34] were also performed. Additionally, Ferreira et al. also performed in vivo and in vitro toxicological assays and concluded that short-term use is safe [8,28].

The anti-inflammatory evaluation of the *G. tridentata* extracts and mainly those reports which were recently achieved [8,31,35] will be the focus of this review. In the most recent evaluations, the authors tested parts of the plant separately and established that the anti-inflammatory effects of plant extracts could occur through different mechanisms. Moreover, the roots, which are not used in traditional medicine, also presented strong anti-inflammatory activity [8]. Likewise, the antioxidant and anti-inflammatory activity is associated with the species richness in polyphenolic compounds, particularly flavonoids.

## 3. Structural Pattern of the Flavonoids Isolated from *Genista tridentata*

Several authors demonstrated that *G. tridentata* produces several flavonoids; these metabolites are those that most contribute to the plant anti-inflammatory activity. Therefore, herein the flavonoids that were isolated from *G. tridentata* extracts or identified in will be discussed.

**Figure 1:**
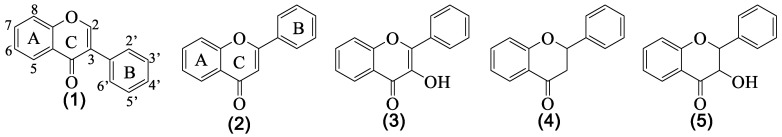
Structure of the classes of flavonoid derivatives found in *G. tridentata*.

From the several established profiles, it is evident that the only classes of flavonoids detected were isoflavones **1**, flavones **2**, flavonols **3**, flavanones **4** and flavanonols **5** (Figure 1), and the major ones are isoflavones and flavonols (Table 2 and Table 3).

**Figure 2:**
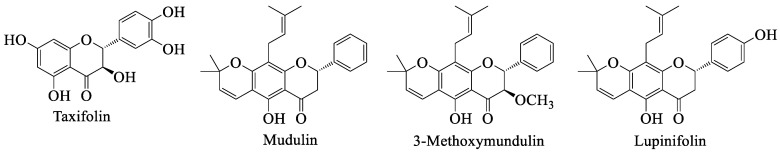
Structure of some flavonoid derivatives found in *G. tridentata*.

As far as we could find, the first report on the *G. tridentata* flavonoids allowed the isolation of four isoflavone derivatives **1a** to **1d**, and one flavonol **3a** (Table 2 and Table 3) [20]. Four years later, the same research group found two other isoflavones **1e** and **1f**, and flavonols **3b** and **3c**, (Table 2 and Table 3) [36]. The first flavone derivatives were just reported in 2012 and were luteolin derivatives **2a** and **2b** (Table 2) [28]. Flavanonols were just uncovered, for the first time, in 2014 and are taxifolin derivatives, whereas flavanone derivatives were only reported in 2020 (Figure 2) [8,29].

Our literature survey showed that the compounds indicated in Table 2 were found by several authors, with the exception of isoflavones **1i** and **1j**, and all the flavone derivatives that were just reported once [8,28,33]. Furthermore, through the analysis of Table 2, it is possible to detect that most of the flavonoids present one or more hydroxy groups and almost all are linked to saccharide units. Usually, this substitution pattern is associated with the anti-inflammatory property of a flavonoid [37].

It is important to complement the information listed in Table 2 with the information that other isoflavone glycosides were described, namely biochanin A hexoside [8,29,31,33] and genistein hexoside [8,31], but the authors did not identify the hexose nor its position in the isoflavone ring. There are also references describing the presence of a methylbiochanin A or a methylprunetin [8,29,30,31] derivative. In all of these cases, although this is important information about the *G. tridentata* profile, it was not included in Table 2 because its structure is not fully established. This suggests that some investment in phytochemical studies involving *G. tridentata* extracts is still needed.

The substitution pattern of flavanonol derivatives includes several hydroxy groups and glucosides, as well as a disaccharide unit. The most referred derivatives were isoquercitrin **3a** and rutin **3c** (Table 3), and again we are in the presence of compounds having the required substitution pattern for being promising anti-inflammatory agents [37]. Additionally, quercetin hexoside derivatives were also found, but the authors were again unable to identify the hexose or its position [29,30,31].

Finally, we can find the identification and isolation of flavanonols and flavanones (Figure 2). Some authors have reported the presence of taxifolin [33] or its glucosides [8], whereas others just mention hexoside derivatives [29,30,31]. One fact is consistent—*G. tridentata* produces taxifolin derivatives. The last examples were recently reported and apart from being slightly different, they were isolated from the plant roots [8], which is also uncommon due to the fact that most of the works were performed using flowers or aerial parts. This highlights that some parts of the plant should still be studied.

## 4. Flavonoids with Anti-Inflammatory Activity

In the previous section, we showed the richness of at *G. tridentata* in flavonoids; additionally, the major class, that is isoflavones, is commonly associated with beneficial anti-inflammatory properties [10]. Yu et al. discussed, in their excellent review [10], the possible isoflavones anti-inflammatory mechanisms, of which herein we highlight the main points (Table 4). Still, we suggest that our readers consult the original review for details. According to the authors, isoflavones may be involved in the scavenging of reactive oxygen species and, in doing so, they prevent the production of peroxynitrite, species that can oxidize low-density lipoproteins. With this effect, isoflavones can prevent cell membrane damage. However, they can also act by inhibiting the production of pro-inflammatory cytokines and chemokine species such as *IL-1β*, *IL-6*, *IL-12* and *TNF-α*, or by inhibiting pro-inflammatory enzymes, such as cyclooxygenase, nitric oxide synthases, lipoxygenase and phospholipase A2, enzymes involved in the production of inflammatory mediators. Finally, there is also evidence that isoflavones can be involved in the regulation of *NF-κB* factor signaling and, through that regulation, decrease the production of pro-inflammatory cytokines (Table 4) [10,38,39].

Spagnuolo et al. discussed the flavonoids neuroprotective potential, in particular flavonols, another family well represented in *G. tridentata* [40]. There is some evidence, at least in in vitro studies, that these flavonoids reduce neuroinflammation also by regulating important signaling pathways such as *NF-κB* and MAPKs (Table 4) [40].

Considering all these pieces of evidence and the fact that several flavonoids were found in *G. tridentata*, we selected some significative examples to discuss their anti-inflammatory potential, and Table 4 summarizes the effect and mechanism of action of the selected flavonoids.

### 4.1. Biochanin A and Prunetin

**Figure 3:**
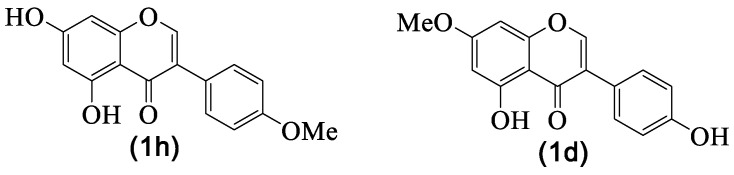
Biochanin A **1h** and prunetin **1d** structures.

Biochanin A **1h** and prunetin **1d** are isomeric natural isoflavones (Figure 3) produced by *G. tridentata* not as the major components, but in small amounts, 4.8% (μg/g) for biochanin A **1h** and 4.1% (μg/g) for prunetin **1d** [29]. Some derivatives are also reported, and in particular, the methyl derivative that was not fully identified [29]; in fact, if there is no evidence of mass spectra fragments containing the characteristic A ring fragment [8] or the compound was isolated [20], it is possible to confuse these isomers. One fact is consistent—*G. tridentata* produced one or both.

As far as we could find, prunetin **1d** was isolated for the first time in 1952 from *Pterocarpus angolensis* DC. [85] and biochanin A **1h** was isolated from *Cicer arietinum* L. in 1945 [86]. Although these isoflavones’ natural occurrence seems to be similar, from the biological evaluation point of view, biochanin A **1h** has been extensively studied, and several health benefits were attributed to its consumption as well as its possible use to develop new drugs [87,88], and anti-inflammatory activity is among those biological properties.

In this century, several evaluations regarding the biochanin A **1h** anti-inflammatory activity have been performed (Table 4), and the first example is the study of Kalayciyan et al. [41], in which the compound potential to treat the Behçet’s disease was established. The main anti-inflammatory effect of the compound is to decrease the secretion of interleukin-8 (*IL-8*), a potent leukocyte chemotactic factor known to induce inflammation [41]. More recently, it was also proved that biochanin A **1h** inhibits the *IL-8* expression in lipopolysaccharides (LPS)-stimulated human vascular endothelial cells in a dose-dependent manner [42], as well as in focal cerebral ischemia/reperfusion in rats [43]. The biochanin A **1h** effects on other interleukins levels, such as *IL-1β*, *IL-6*, *IL-10*, and *IL-18*, were evaluated in the last feew years, with *IL-1β* being the most studied one [44,45,46,47,48,49,50,51,52,53,54]. All these studies proved the inhibitory effect that biochanin A **1h** has on these inflammatory cytokines. However, the most important aspect is the fact that some of the studies were performed in vivo [43,46,49,50,51,52], which is a forward step to establish this compound pharmacological potential.

The inhibition of another important pro-inflammatory species, such as TNF-α, was also evaluated by several authors [42,43,44,45,46,47,48,49,52,53,54,55,56], as well as the inhibiting pro-inflammatory enzymes [49,55] and key phosphorylation steps [44,48,56,57]. All of these studies suggested that biochanin A **1h**’s anti-inflammatory effect occurs by suppressing the pathways NF-κB and MAPK [53,56,57,58], but is also associated with the up-regulation of PPAR expression [43,45,53,54]. Prunetin **1d**, a much less studied compound, also presents potent in vitro [56,59,60] and in vivo [59] anti-inflammatory activity, and apparently, its mechanism of action is also associated with the inhibition of the NF-κB pathway [59].

It should be highlighted that several of the studies mentioned above included the evaluation of cytotoxic effects, and all demonstrated that both isoflavones do not affect the viability of the cells, and in the subsequent tests the authors used noncytotoxic concentrations. From these studies, essential facts arose—prunetin **1d** should be subjected to more evaluations. Moreover, pharmacodynamic and pharmacokinetic parameters of both isoflavones should be evaluated in order to implement some clinical trials in the future.

### 4.2. Daidzein

**Figure 4:**
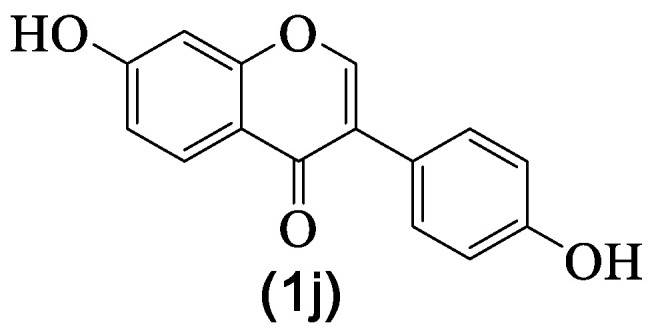
Daidzein **1j** structure.

Daidzein **1j** (Figure 4) is a natural isoflavone with a significant occurrence, mainly in fruits and nuts [89], which is the reason why humans are exposed to it and also to its health benefits [90]. In fact, several pharmacological properties are attributed to this isoflavone [91], including anti-inflammatory potential [10,91]. Although daidzein **1j** occurrence in *G. tridentata* is rare, only one report on its identification was reported (Table 2), we decided to include here the most recent works on its anti-inflammatory activity, since its occurrence seems to be exclusively in the plant roots [8]. This fact gives importance to that part of the plant, while importance usually is only given to the flowers and aerial parts, which are the ones used traditionally.

The most recent studies involved in vivo studies with daidzein **1j**—the reasons why are herein highlighted. Due to its occurrence in common fruits [89], daidzein **1j** is present in mankind’s diet, and it is a nontoxic compound [52]. These recent studies confirmed daidzein **1j**’s strong anti-inflammatory activity as well as settling on its mechanism of action (Table 4). Mainly, daidzein **1j** strongly affects various pathways, including NF-κB, p38MAPK, and TGF-β1. Regardless of this potential as an anti-inflammatory drug, as far as we could find, daidzein **1j** is not involved in clinical trials.

### 4.3. Genistein

**Figure 5:**
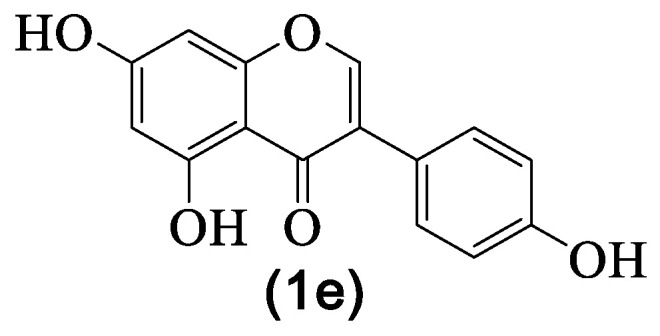
Genistein **1e** structure.

Genistein **1e** (Figure 5), like daidzein **1j**, occurs naturally in everyday food, such as fruits and nuts [89], and as far as we could find, it is non-toxic for humans [92], which was also recently reinforced by Kumar et al. [93]. The pharmacological potential of genistein **1e** is well documented [94]; more recently, an overview regarding their mechanism of action in cancer models was published [95], and in some aspects, the anticancer and the anti-inflammatory activities are associated.

Regarding the anti-inflammatory activity studies, it should be emphasized that, recently, there are more in vivo studies, meaning that scientists are interested in giving this natural isoflavone new medicinal applications. From the reported results, we select a few (Table 4) that demonstrategenistein **1e**’s potential to become an anti-inflammatory drug.

It can be seen that like the isoflavones mentioned above, genistein **1e** targets the same pathways, with an emphasis on the upregulation of the PPARγ signaling pathway and downregulation of the NF-κB signaling pathway, as well as the decrease in several inflammatory mediators (Table 4). In light of the referred studies, genistein **1e** is a candidate to be used in the prevention or treatment of inflammation-related diseases. For example, it could be used to target microRNAs, which is considered a therapeutic target for liver disease. In fact, the results show that the anti-inflammatory activity of genistein **1e** downregulated microRNA expression of liver inflammation [70] but also pro-inflammatory cytokines species such as *IL-1β* and *TNF-α* [70,73]. Another interesting example is its ability to attenuate NF-κB inflammatory signaling in the brain with consequent inhibition of pro-inflammatory cytokines release, which gives genistein **1e** the possibility to become a new drug able to relieve chronic sleep deprivation’s adverse effects [71]. Furthermore, there is some evidence supporting that genistein **1e** can, through its anti-inflammatory activity, prevent cardiovascular diseases [74]. Altogether, these findings suggest that genistein is a good candidate for future clinical trials.

### 4.4. Rutin

**Figure 6:**
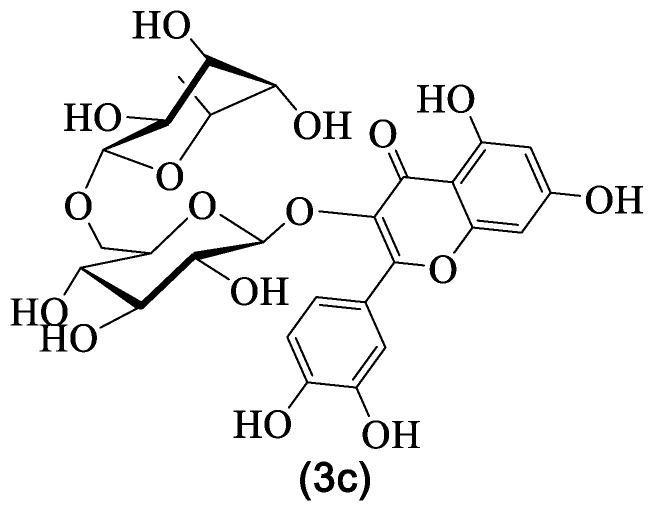
Rutin **3c** structure.

Rutin **3c** (Figure 6) is amongst the most found flavonoids in *G. tridentata* (Table 3), for which several biological and pharmacological properties have been established and reviewed through the years [96,97,98,99,100]. Some more specific activities, such as antidiabetic effects [101], reestablishment of the immune homeostasis [96,102], neuroprotective effects [98,103,104] and anticancer effects [98,99,105] were also addressed. Furthermore, some toxicological studies were also performed [98,106] as well as pharmacokinetic [98], bioavailability [99] and formulation development [100]. It should be emphasized that the mentioned properties prompted some clinical trials using rutin **3c** [107,108] and although the results are not remarkable, they at least confirm that it is safe to use rutin **3c**.

Obviously, rutin **3c**‘s anti-inflammatory activity was also evaluated and several interesting results were reported (Table 4). It is known that in general, flavonoids decrease the production of pro-inflammatory interleukins, mainly IL-1β, IL-6, and IL-8, but also tumor necrosis factor α (TNF-α). There is evidence that rutin **3c** anti-inflammatory mechanism also involves the downregulation of these pro-inflammatory species [76,77,78,79,80]. The results show that rutin **3c** can also exert its anti-inflammatory activity through other mechanisms (Table 4), from which can be highlighted the inhibition of the HMGB1 signaling pathway through the downregulation of TLR4 and RAGE expressions [75] and also the inhibition of the MPO activity [80]. The last one is an important example because it provides evidence that rutin **3c** can be a possible therapeutic agent for autoimmune diseases [80].

Collectively, the results demonstrate that rutin **3c** attenuates inflammation through several mechanisms and is a nontoxic compound, so clinical trials more focused on its anti-inflammatory potential should be implemented. In this regard, Kalita and Das [109] studied the efficiency of a rutin **3c** formulation to be used in the treatment of inflammations through the long-term delivery via the skin. Their results, although preliminary, are sufficiently good to encourage future investigations.

### 4.5. Taxifolin

Our last example is taxifolin (Figure 2), which, as shown in the previous section, occurs in *G. tridentata,* mainly linked to sugar moieties. Nevertheless, we specify here some interesting studies due to the fact that in a living organism, it is possible to obtain the aglycone. The taxifolin anti-inflammatory potential has been known, at least, since 1971 [110] and recently Sunil and Xu published an interesting review on taxifolin’s health benefits [111]. Some important aspects arose from this review: the first is the broad biological potential of taxifolin, mainly using in vitro evaluations, but also that the anti-inflammatory and toxicological evaluations are still scarce. The few examples (Table 4) suggest that its mechanism of action is similar to the one reported for the other flavonoids, that is also mainly targets the NF-κB and MAPK pathways. Although, the anti-inflammatory assessments are scarce, they suggest taxifolin’s potential to be a drug candidate for the treatment of inflammations, suggesting that it should be further investigated.

## 5. Conclusions

This survey demonstrates beyond any doubt that *G. tridantata* is a source of bioactive metabolites, some of which present interesting anti-inflammatory activities which, in turn, contribute to the extracts’ anti-inflammatory activity. Amongst our findings, the toxicological evaluations of both extracts and pure compounds are important and contribute to establishing *G. tridentata*’s medicinal value as well as the secondary metabolites’ pharmacological value. However, in our opinion, some efforts on the plant taxonomy should be made to prevent confusion in the data reported. Moreover, we think that an extra effort on clinical trials, mainly concerning the pure compounds used as drugs, should be performed.

## Figures and Tables

**Table 1:** Biological assays of *Genista tridentata* extracts.

Plant Part	Solvent	Activity Tested	Method	Ref.
Aerial parts	Ethanol and water	Antioxidant (ethanol, IC_50_ = 60.39 ± 1.79 μg/mL; water, IC_50_ = 42.97 ± 1.69 μg/mL)	DPPH scavenging β-Carotene bleaching test	[24]
Flowers, stems and leaves	Methanol	Antioxidant (flowers, IC_50_ = 26.1 ± 1.3 mg/L; stems and leaves, IC_50_ = 69.7 ± 11.9 mg/L)	DPPH scavenging β-Carotene bleaching test	[25]
Flowers	Methanol	Antioxidant	DPPH scavenging (IC_50_ = 0.15 ± 0.01 mg/mL) β-Carotene bleaching test (IC_50_ = 0.14 ± 0.02 mg/mL) Reducing power (IC_50_ = 0.13 ± 0.00 mg/mL) TBARS inhibition (IC_50_ = 0.12 ± 0.02 mg/mL)	[26]
Flowers and leaves	Hydroethanolic	Antioxidant (flowers, IC_50_ = 1016 mg/L; leaves, IC_50_ = 704 mg/L	DPPH scavenging β-Carotene bleaching test Reducing power ABTS scavenging	[27]
Purchased plant material	Water	Antioxidant (%AA = 169.5 ± 17.2)	β-Carotene bleaching test ABTS scavenging	[28]
Purchased plant material	Methanol	Antioxidant	DPPH scavenging (IC_50_ = 0.18 ± 0.01 mg/mL) β-Carotene bleaching test (IC_50_ = 0.48 ± 0.09 mg/mL) Reducing power (IC_50_ = 0.11 ± 0.00 mg/mL) TBARS inhibition (IC_50_ = 1.18 ± 0.06 mg/mL)	[29]
Purchased plant material	Hot water	Antioxidant	DPPH scavenging (IC_50_ = 50 ± 1 μg/mL) β-Carotene bleaching test (IC_50_ = 266 ± 25 μg/mL) Reducing power (IC_50_ = 105 ± 2 μg/mL) TBARS inhibition (IC_50_ = 93 ± 4 μg/mL)	[30]
Flowers	Hot water	Antioxidant (TABARS, IC_50_ = 8.4 ± 0.2 μg/mL; OxHLIA, IC_50_ = 37.7 ± 0.9 μg/mL)	TBARS inhibition Oxidative haemolysis inhibition	[31]
Flowers	Hydromethanolic	Antifungal (*Candida albicans*, 10 mm inhibition zone; *Candida glabrata*, 11 mm inhibition zone)	Disc diffusion test	[32]
Aerial parts	Hydromethanolic	Antibacterial (*Staphylococcus aureus*, MIC = 39.1 μg/mL)	Microplate bioassay	[33]
Flowers	Hot water	Antimicrobial (*Escherichia coli*, MIC = 0.5 mg/mL; *Salmonela typhimurium*, MIC = 1 mg/mL; *Bacillus cereus*, MIC = 1 mg/mL; *Listeria monocytogenes*, MIC = 1 mg/mL; *Aspergillus niger*, MIC = 8 mg/mL; *Aspergillus versicolor*, MIC = 0.5 mg/mL; *Penicillium funiculosum*, MIC = 0.5 mg/mL; *Penicillium verrucosum*, MIC = 0.5 mg/mL)	Disc diffusion test	[31]
Flowers	Hot water	Cytotoxicity (HeLa, GI_50_ = 242 ± 10 μg/mL; HepG2, GI_50_ = 262 ± 11 μg/mL)	Against tumor cells HeLa, HepG2, MCF-7 and NCi-H460 and non-tumor cells PLP2	[31]
Inflorescences	Hot water	Immunostimulatory (significant activity for 200 μg/mL)	Macrophage cell viability and NO production	[34]
Purchased plant material	Water	Toxicity (non toxic at 375 mg/L)	MTT assay; mitochondrial swelling,	[28]
Flowers, leaves, stems and roots	Ethanol	Toxicity (non toxic at 100 μg/mL)	Resazurin assay	[8]
Flowers	Hot water	Anti-inflammatory (>400 μg/mL)	Determination of LPS-induced NO production by Murine macrophage (RAW 264.7) cell lines	[31]
Flowers, leaves, stems and roots	Ethanol	Anti-inflammatory (significantat 100 μg/mL)	LPS-induced transcription of pro-inflammatory genes *IL-1β, Nos2*, *Ptgs2*, *IL-6*, and *TNF-α*; Western blot analysis	[8,35]

AA, antioxidant activity; GI_50_, values correspond to the concentration that causes 50% inhibition of cell proliferation; IC_50_, values corresponded to the extract concentration that inhibits in 50% the oxidation and inflammatory process; MIC, minimum inhibitory concentration.

**Table 2:** Isoflavones and flavones produced by *G. tridentata*.

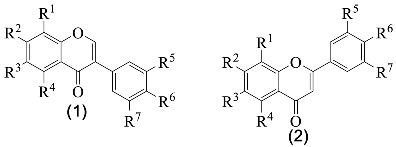									
Nº	Name	R^1^	R^2^	R^3^	R^4^	R^5^	R^6^	R^7^	Ref.
**1a**	Sissotrin	H	OGlc	H	OH	H	OMe	H	[8,20,29,30,36]
**1b**	Genistin	H	OGlc	H	OH	H	OH	H	[20,29,30,33,36]
**1c**	5,5′-Dihydroxy-3′-metoxi- -isoflavone-7-O-β-glucoside	H	OGlc	H	OH	OMe	H	OH	[8,20,29,30,31,36]
**1d**	Prunetin	H	OMe	H	OH	H	OH	H	[8,20,29,30,36]
**1e**	Genistein	H	OH	H	OH	H	OH	H	[8,27,29,30,31,33,36]
**1f**	7-Methylorobol	H	OMe	H	H	OH	OH	H	[29,30,36]
**1g**	Genistein-8-*C*-glucoside	Glc	OH	H	OH	H	OH	H	[29,30,31]
**1h**	Biochanin A	H	OH	H	OH	H	OMe	H	[8,29,30]
**1i**	5-Hydroxy-4′,7-dimethoxy- -isoflavone	H	OMe	H	OH	H	OMe	H	[8]
**1j**	Daidzein	H	OH	H	H	H	OH	H	[8]
**2a**	Luteolin-*O*-glucuronide	H	OGlc	H	OH	OH	OH	H	[28]
**2b**	Luteolin-*O*-(*O*-acetyl)glucuronide	H	OGlcA-Ac	H	OH	OH	OH	H	[28]
**2c**	Apigenin	H	OH	H	OH	H	OH	H	[33]

Glc = glucoside unit; GlcA = glucuronide unit; Ac = acetyl.

**Table 3:** Flavonols produced by *G. tridentata*.

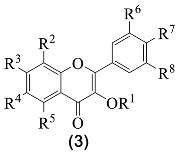										
Nº	Name	R^1^	R^2^	R^3^	R^4^	R^5^	R^6^	R^7^	R^8^	Ref.
**3a**	Isoquercitrin	Glc	H	OH	H	OH	OH	OH	H	[20,29,30,31,33,36]
**3b**	Myricetin-6-*C*-glucoside	H	H	OH	Glc	OH	H	OH	OH	[8,29,30,36]
**3c**	Rutin	Rha-Glc	H	OH	H	OH	OH	OH	H	[29,30,31,33,36]
**3d**	Isorhamnetin-*O*-glucoside	Glc	H	OH	H	OH	OMe	OH	H	[28]
**3e**	Myricetin-3,4′-di-*O*- -glucoside	Glc	H	OH	H	OH	OH	OGlc	OH	[28]
**3f**	Astragalin	Glc	H	OH	H	OH	H	OH	H	[8]
**3g**	Isorhamnetin-3-*O*- -glucoside	Glc	H	OH	H	OH	OMe	OH	H	[8]
**3h**	Kaempferol	H	H	OH	H	OH	H	OH	H	[8]

Glc = glucoside unit; Rha = rhamnoside unit.

**Table 4:** Anti-inflammatory effects of the selected flavonoids.

Flavonoid	Model	Mechanisms
Biochanin A	In vitro: cytokine release from keratinocytes and HMEC-1 endothelial cells in serum from patients with Behçet’s disease [41] In vitro: LPS-induced inflammation in HUVED cells [42] In vivo: focal cerebral ischemia–reperfusion model [43] In vitro: LPS-induced pro-inflammatory responses in murine BV2 microglial cells [44] In vitro: LPS-induced inflammatory cytokines and mediators production in murine BV2 microglial cells [45] In vivo: LPS/GalN-induced liver injury [46] Ex vivo: interleukin-1β-induced catabolic inflammation through the modulation of NFκB cellular signaling in primary rat chondrocytes [47] In vitro and in vivo: LPS-induced damage of dopaminergic neurons [48] In vivo: cisplatin induced acute kidney injury in mice [49] In vivo: ritonavir induced hepatotoxicity [50] In vivo: transient coronary ligation in Sprague-Dawley rats [51] In vivo: LPS-induced acute lung injury in mice [52] In vitro: LPS-induced NO production, LPS-induced IKK activity, LPS-induced phosphorylation of IκBα and p38 MAPK [53] In vitro: CCl_4_-induced hepatotoxicity in rats [54] In vivo: Sprague-Dawley rat subarachnoid hemorrhage [55] In vitro: barrier function of intestinal epithelial CaCo-2/TC-7 cells via TEER measurements [56] In vitro: LPS-stimulated macrophages [57] In vivo: focal cerebral ischemia established by middle cerebral artery occlusion [58]	↓IL-8 ↓IL-8, TNF-α, VCAM-1, ICAM-1, E-selection ↑PPAR-γ ↓IL-8, TNF-α, P38 expression ↓IL-1β, TNF-α, NO, phosphorylation of JNK, ERK and p38 ↓IL-1β, TNF-α, NO, PGE_2_, NF-κB ↑PPAR-γ IL-1β, TNF-α, ALT, AST, MDA, TXNIP, NLRP3 inflammasome ↑SOD, GPx, catalase, HO-1, Nrf2 ↓IL-1β, TNF-α, IL-6, IL-1α, INFγ, IL- 2, GM-CSF, fractalkine, MCP-1, MIP-3α, LIX ↓IL-1β, TNF-α, IL-6, phosphorylation of JNK, ERK and p38, ↓IL-1β, TNF-α, caspase-3, p53 protein ↓IL-1β, IL-6 ↑IL-10 ↓IL-1β, IL-18, IL-6, TNF-α IL-1β, IL-6, TNF-α, TLR4/NF-κB ↑PPAR-γ IL-6, TNF-α PPAR-γ, PPAR-α iNOS, COX2, TNF-α sTNFR1, TNF-α, NF-κB, ERK, tyrosine phosphorylation ↑SOD, GSH-Px, HO-1, Nrf2 ↓iNOS, phosphorylation of IκBα and p38 MAPK ↓TLR/NF-κB
Prunetin	In vitro: barrier function of intestinal epithelial CaCo-2/TC-7 cells via TEER measurements [56] In vitro: LPS-stimulated RAW 264.7 macrophage [59] In vivo: LPS-induced septic shock [59] In vitro: LPS-induced in- flammatory response and MUC5AC expression [60]	↓sTNFR1, TNF-α, NF-κB, ERK, tyrosine phosphorylation ↓iNOS, PGE2, COX2, NF-κB, p38, IL-1β, TNF-α IL-1β, TNF-α IL-8, IL-6, MUC5AC, TLR4/MyD88
Daidzein	In vitro: LPS-stimulated macrophages [57] In vivo: angiotensin II-induced AAA [61] In vivo: 5-fluorouracil-induced intestinal mucositis [62] In vivo: cisplatin-induced kidney injury [63] In vivo: ischemia/reperfusion injury-induced neurological function deficits in Sprague-Dawley [64]	↓IL-6 ↓IL-1β, TNF-α, NF-κB, iNOS, COX-2, p38MAPK, TGF-β1 ↓IL-1β, IL-6, TNF-α, NO, COX-2 ↓IL-6, TNF-α, MDA, NO, COX-2, MAPK ↑SOD, GSH ↓TNF-α, NF-κB subunit p65
Genistein	In vitro: LPS-stimulated macrophages [57] In vitro: homocysteine-induced endothelial cell inflammation [65] In vivo: cyclophosphamide - induced hepatotoxicity [66] In vivo: LPS-induced microglial activation in murine BV2 microglial cell line and primary microglial culture [67] In vivo: imiquimod- induced psoriasis-like lesions in mice [68] In vivo: DSS-induced murine colitis [69] In vivo: NASH mouse model [70] In vivo: chronic sleep deprivation [71] In vitro: barrier function of intestinal epithelial CaCo-2/TC-7 cells via TEER measurements [56] In vivo: mouse model of periodontitis [72] In vivo: high-fat high-fructose diet-induced NASH rats [73] In vitro: angiotensin II-stimulated CRP and MMP-9 expression in VSMC [74]	↓IL-6, TNF-α PPAR-γ, PPAR-α NF-κB subunit p65, IL-6, ICAM-1 ↓IL-1β, COX-2, MPO ↓IL-1β, IL-6, COX-2, iNOS, TNF-α, NF-κB, MAPK ↓IL-1β, IL-6, IL-8, TNF-α, IL-17, IL-23, CCL2, NF-κB, VEGFA ↓IL-1β, IL-18, TNF-α, MPO, NLRP3 inflammasome ↓IL-6, TNF-α, ↓IL-1β, IL-6, COX-2, iNOS, TNF-α, NF-κB p65 ↑HO-1, Nrf2 ↓sTNFR1, tyrosine phosphorylation ↓TNF-α, COX-2, Nos2, ICAM-1, MMP-2, MMP-9 ↓TNF-α, NF-κB ↓p-ERK1/2, p-p38, NF-κB ↑PPAR-γ,
Rutin	In vivo: HMGB1-induced inflammation and CLP-induced sepsis model [75] In vivo: LPS-induced acute endotoxemic kidney injury in C57BL/6 mice [76] In vivo: NaF-induced neurotoxicity [77] In vivo: HgCl_2_-induced nephrotoxicity [78] In vivo: HgCl_2_-induced hepatotoxicity [79] In vitro: PMA-induced neutrophil stimulation [80]	↓TLR 4, RAGE, p38 MAPK, VCAM-1, ICAM-1, ERK1/2, NF-κB ↓TLR 4, COX-2, TNF-α, IL-6, SIRT1, NF-κB ↓IL-1β, IL-6, TNF-α ↓IL-1β, IL-33, TNF-α, NF-κB, Bcl-3 ↓IL-1β, TNF-α, NF-κB, Bcl-3, Bcl-2, Bax, p53, p38 MAPK, caspase-3 ↓NO, TNF-α, MPO
Taxifolin	In vitro: osteoclastogenesis [81] In vivo: and ovariectomy-induced osteoporosis [81] In vivo: osteolysis model [82] In vitro: on IgE/Ag-stimulated mast cells including BMMCs [83] In vivo: acetaminophen-induced liver injury [84]	↓AKT, RANKL ↓TNF-α, IL-1β, NF-κB, MAPK, NFATc1, MMP-9, cathepsin K, TRAP ↓MAPK, p38, ERK, JNK; RANKL, NF-κB ↓LTC_4_, IL-6, COX-2, TNF-α, NF-κB ↓ inhibiting metabolic activation mediated by CYP450 enzymes

## Abbreviations

AAA	abdominal aortic aneurysm
ABTS	2,2′-azino-bis(3-ethylbenzothiazoline-6-sulfonic acid
Ag	antigen
AKT	serine/threonine kinase
Bax	Bcl-2 associated X protein
Bcl-2	B-cell lymphoma-2
Bcl-3	B-cell lymphoma-3
BMMCs	bone marrow derived mast cells
caspase-3	cysteine aspartate specific protease-3
CCL2	chemokine ligand 2
CLP	cecal ligation and puncture
CRP	C-reactive protein
CXC	α-chemokines
CYP450	Cytochrome P450
DPPH^●^	2,2-diphenyl-1-picrylhydrazyl radical
DSS	dextran sulfate sodium
E-selection	endothelial cells
ERK	extracellular signal-regulated protein kinase
*G.*	*Genista*
GalN	D-galactosamine
GM-CSF	granulocyte-macrophage colony-stimulating factor
GPx	glutathione peroxidase
HMEC-1	human dermal microvascular endothelial cell-1
HMGB1	high mobility group box 1
HO-1	heme oxygenase-1
HUVEC	human umbilical vein endothelial
ICAM-1	intercellular adhesion molecule-1
IFNγ	interferon gamma
IgE	immunoglobulin E
IKK	IκB kinase
IL-10	interleukin-10
IL-12	interleukin-12
IL-18	interleukin-18
IL-1α	interleukin-1α
IL-1β	interleukin-1β
IL-2	interleukin-2
IL-6	interleukin-6
IL-8	interleukin-8
iNOS	inducible nitric oxide synthase
JNK	c-jun *N*-terminal kinase
LIX	lipopolysaccharide-induced CXC chemokine
LPS	lipopolysaccharides
LTC_4_	cysteinyl leukotriene 4
MAPK	mitogen-activated protein kinases
MCP-1	monocyte chemoattractant protein-1
MDA	malondialdehyde
MIP-3α	macrophage inflammatory protein 3 α
MMP	matrix metalloproteinases
MPO	myeloperoxidase
MTT	3-(4,5-dimethylthiazol-2-yl)-2,5-diphenyltetrazolium bromide
MUC5AC	mucin 5AC glycoprotein
MyD88	myeloid differentiation primary response 88
NASH	nonalcoholic steatohepatitis
NF-κB	nuclear factor kappa-light-chain-enhancer of activated B cells
NFATc1	nuclear factor-activated T cells c1
NLRP3	NRL pyrin domain containing 3
Nos2	nitric oxide synthase 2
Nrf2	nuclear factor erythroid 2
NRL	nucleotide-binding, leucine-rich repeat containing proteins
PGE_2_	prostaglandin E2
PMA	phorbol 12-myristate 13-acetate
PPAR-γ	peroxisome proliferator-activated receptor gamma
Ptgs2	prostaglandin-endoperoxide synthase 2
RAGE	receptor for advanced glycation end-products
RANKL	receptor activator of nuclear factor-κB ligand
SIRT1	sirtuin 1
SOD	superoxide dismutase
sTNFR1	soluble tumor necrosis factor receptor-1
TBARS	thiobarbituric acid reactive substances
TEER	transepithelial electrical resistance
TGF-β1	transforming growth factor β1
TLR 4	toll-like receptors 4
TNF-α	tumor necrosis factor alpha
TRAP	tartrate-resistant acid phospha- tase
TXNIP	thioredoxin-interacting protein
VCAM-1	vascular cytoadhesion molecule-1
VEGF	vascular endothelial growth factor
VEGFA	vascular endothelial growth factor A
VSMC	vascular smooth muscle cells
